# Strategic menu optimization could reduce carbon emissions and saturated fat consumption: a simulation modelling study of UK hospital inpatient meals

**DOI:** 10.1098/rstb.2024.0152

**Published:** 2025-09-18

**Authors:** Annika N. Flynn, Taro Takahashi, Jeffrey M. Brunstrom

**Affiliations:** ^1^ Nutrition and Behaviour Unit, School of Psychological Science, University of Bristol, Bristol BS8 1TU, UK; ^2^ Bristol Veterinary School, University of Bristol, Langford BS40 5DU, UK; ^3^ Agri-Food and Biosciences Institute, Hillsborough BT26 6DR, UK; ^4^ NIHR Bristol Biomedical Research Centre: Diet and Physical Activity Theme, University Hospitals Bristol and Weston NHS Foundation Trust and University of Bristol, Bristol BS8 2BN, UK

**Keywords:** sustainability, dietary intake, menu design, population health, carbon footprint, saturated fatty acid

## Abstract

Interventions to improve nutritional quality and environmental sustainability often rely on ‘nudging’, education or (dis-)incentive-based measures. As part of the Transforming UK Food Systems Programme (Flynn *et al*., 2025), we recently proposed a ‘fourth’ approach that complements these strategies—whereby dishes are swapped across a weekly menu to alter daily inter-dish competition, and thus choice architecture—and validated its effectiveness in a university canteen where meals are pre-paid for the whole year. As a second step in assessing the potential of our approach, we modelled strategic menu swaps in an alternative public procurement setting. Eleven weekly inpatient menus were sampled opportunistically from National Health Service hospitals across the UK and combined with responses from an online food-choice task (*n* = 550, 50 participants per region). Expected reductions in weekly carbon footprint and saturated fatty acid (SFA) intake were then calculated under mathematically optimized menus, targeting each outcome independently and simultaneously. Targeting a single variable resulted in a 12.7–29.3% reduction in carbon footprint and a 6.5–31.5% reduction in SFA intake. Joint optimization achieved a 9.1–29.3% and a 5.0–26.5% reduction, respectively. We discuss key next steps for real-world implementation in hospitals and other catered environments such as schools.

This article is part of the theme issue ‘Transforming terrestrial food systems for human and planetary health’.

## 1. Introduction

Efforts to change food consumption behaviour almost always focus on either ‘nudging’, education or (dis-)incentive-based measures via legislation [1,2]. While these approaches have been shown to promote healthy and environmentally sustainable food choices [3,4], the extent to which permanent and meaningful behaviour change can be achieved remains unclear [3,5]. Moreover, there may be some environments where traditional interventions are less effective or even inappropriate. For example, in hospitals and care homes, patients and residents, given their circumstances, may be more concerned with food enjoyment than sustainability.

**Figure 1.**
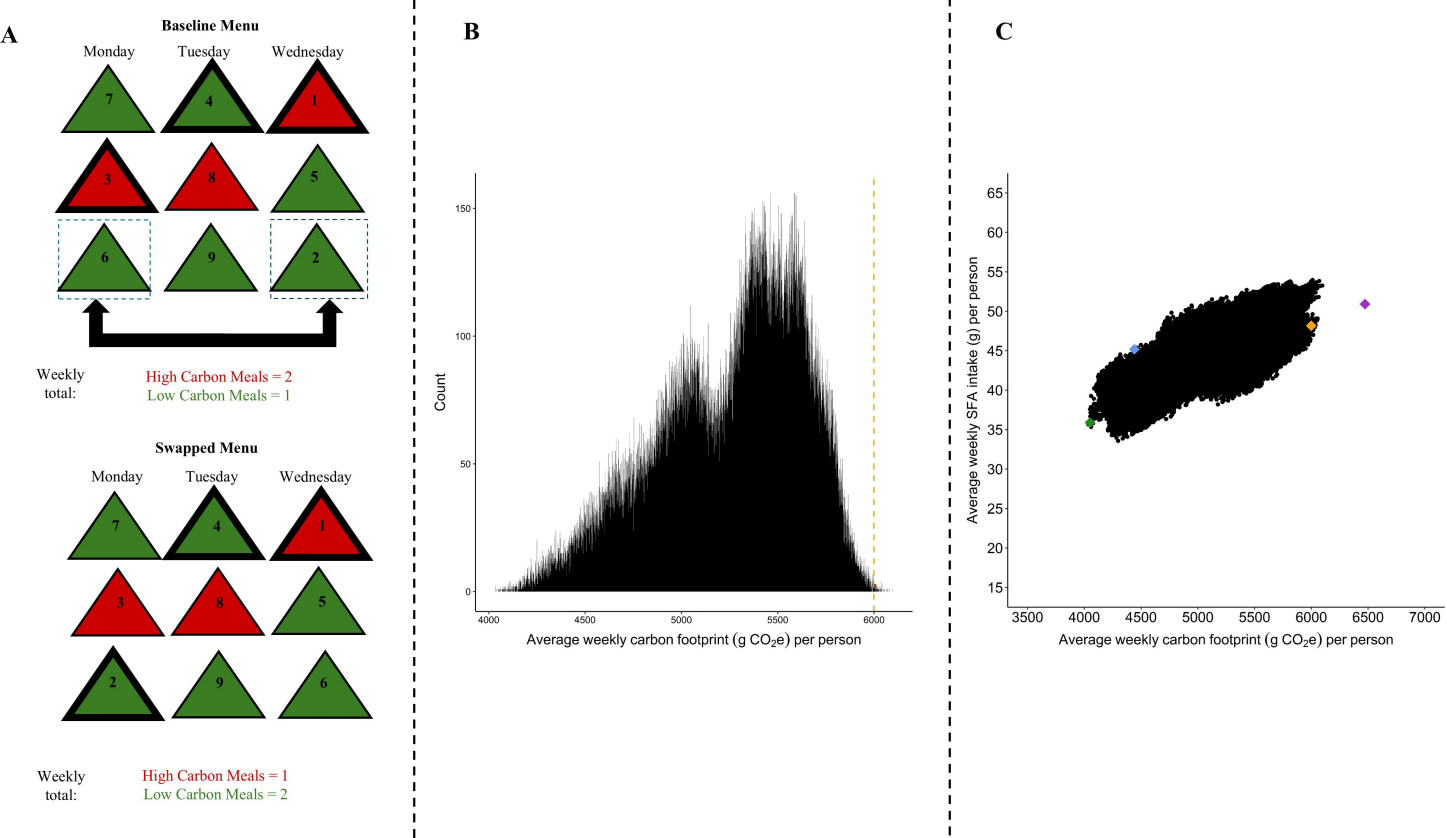
Panel A shows two hypothetical menu scenarios. In both, the red triangles depict high carbon footprint dishes and the green triangles indicate the converse. The number in each triangle represents a single person’s ranked meal preference (1 = most preferred, 9 = least preferred). In the baseline menu scenario, assuming that the most preferred dish is selected each day, the third, fourth and topmost preferred dishes will be selected on Monday, Tuesday and Wednesday, respectively (indicated with a thicker border). Hence, two-thirds of the chosen dishes have a high carbon footprint. The swapped menu scenario shows the effects of swapping the two dishes highlighted by dotted rectangles in the baseline menu. Because swapping changes the competition structure within each day, the person now chooses only one high-carbon dish. In this example, two green dishes have been swapped, yet this causes a 50% reduction in high-carbon meals. Note, this panel depicts the effect of a simple single menu-item swap. Across 5 days, with 3 options offered per day, there are ~1.4 million unique menu combinations that can be considered. Panel B builds on Panel A and depicts the effects of menu-item swapping across 5 days with 3 options per day on average weekly carbon footprint (g CO_2_e) per person from a previous study [6]. Here, the histogram shows the number of different swapped menu combinations (*n* = 113,400) and their respective carbon footprint. The dashed yellow line indicates the predicted average weekly carbon footprint (g CO_2_e) per person associated with the baseline menu. Panel C builds on Panel B and illustrates the effects of menu-item swapping across two outcomes of interest, carbon footprint (*x*-axis) and intake of saturated fatty acid (SFA) (*y*-axis) in a previously conducted study. Here, every dot indicates a unique menu combination (*n* = 113,400). The yellow and green dots indicate the predicted values associated with the baseline and swapped menus, respectively. The purple dot indicates the actual values observed for the baseline menu, while the blue dot indicates the actual values observed for the swapped menu. Our strategic menu-item swapping generated a 31.4% reduction in carbon footprint and an 11.3% SFA intake [6]. Reproduced under Creative Commons Attribution licence (http://creativecommons.org/licenses/by/4.0/).

In a recent study under the Transforming UK Food Systems (TUKFS) Programme, we demonstrated the feasibility of an entirely novel approach that does not rely on any of the three conventional measures highlighted above [6]. Briefly, many people routinely dine in a canteen environment where different meal options are presented each day. For example, 42% of workers in the UK report eating a meal in a canteen [7]. Because each diner can only select one option per mealtime, the weekly impact of diners' choices on environmental and nutritional outcomes depends as much on the menu options *selected* as those *not selected*. For instance, the selection probability of a high-carbon dish is not only influenced by its absolute popularity but also by the relative popularity of a low-carbon dish that is served on the same day. Thus, competition between menu items matters, and, by changing the competition structure between dishes (i.e. choice architecture), we can influence the frequency with which each dish is ultimately selected. As an example, when three main-dish options are offered on each weekday (i.e. 15 unique dishes across a week), there are approximately 1.4 million unique weekly menu configurations, each with a unique competition structure. Thus, by predicting likely choice outcomes for each of these potential menus, it is possible to strategically select one that is expected to deliver the greatest reduction (or enhancement) in a variable of interest, such as carbon footprint (see figure 1 for a visualization of this approach).

In the aforementioned proof-of-concept study, we implemented strategically designed menus in a university hall of residence (*n* ~ 300 students) and then observed actual reductions in carbon footprint and SFA intake of ~31% and ~6%, respectively. Importantly, these reductions were real (not modelled) and they were achieved without modifying or withdrawing any dish from the original menu, and with little impact on consumer satisfaction [6].

Because our intervention does not require recipe reformulation, we see the potential to deliver benefits across several key sectors beyond a university setting. Hence, as the second step towards a future large-scale rollout, here, we sought to evaluate the approach’s potential in an alternative public procurement setting, namely, National Health Service (NHS) hospitals across the UK. We selected the health sector as a case study because there is an urgent need to improve the nutritional quality and sustainability of hospital meals [8], and because patients may be less receptive to education or other behaviour-change interventions because their priority is likely health and recovery. We further note that hospitals are the second largest public-sector provider of catered meals in the UK [9].

We analysed inpatient menu data collected from 11 hospitals and trusts (hereafter ‘hospitals’) and, for each hospital, combined menu data with responses from an online food-choice task to model potential reductions in carbon footprint (g CO_2_e) and SFA intake (g). In this simulation modelling, we chose these target variables to ensure that the results can be compared directly with our earlier study [6]. In this study, we chose carbon footprint because it is a key contributor to global warming [10–12] and SFA intake because it is an important risk factor for a range of negative health outcomes, including cardiovascular disease [13–15]. We also note that there is increasing recognition that health and sustainability goals are not independent [16–18] and, as such, modifications to our food system need to combine both in tandem [12]. In this regard, carbon footprint and SFA intake are not necessarily strongly correlated at the individual dish level [6], so their joint minimization provides an interesting case study.

Given that we are in the gradual process of out-scaling and up-scaling, it is important to acknowledge the scope, objectives and limitations of the work presented here. Producing a full population-level assessment of the costs and benefits of implementing our approach—a step required immediately prior to large-scale rollout—would necessitate a detailed understanding of barriers to, and enablers of, practical implementation, including the impact on procurement logistics, food costs and stakeholder perceptions. Moreover, our optimization algorithm uses preference data to predict how menu-item swapping alters food choice. Since we have yet to address the challenge of collecting meal preference data from actual inpatients, we relied on preference data from carefully selected non-patients (details below); as such, a real-world implementation of our approach would be premature at this point in time.

In preparation for this next step, here, our objective was to assess whether it is even possible to model hospital data. More specifically, our starting questions were: (1) are UK hospital menus structurally suited to our modelling approach and, if so, (2) what level of predicted reductions might be expected? To this end, weighing the need to obtain early answers to these questions against longer-term practical challenges, including the acquisition of patient preference and recipe data, we made three pragmatic decisions through which our results should be interpreted. These decisions and their associated justifications are highlighted below and their limitations are outlined in more detail in §4b:

— In our previous study [6], we focused on menus for evening meals served on weekdays (Monday–Friday) in a university hall of residence. In contrast, hospitals also serve meals at breakfast and lunchtime, and 7 days a week. To make our findings directly comparable with those in our previous work, we only applied our modelling to weekday menus serving the main meal of the day (this timing varies depending on the hospital). Also, in line with our previous study, side dishes were excluded from our optimization and we only collected preference data from omnivores.
— Our approach relies on knowing the carbon footprint and SFA content of each dish. As none of the hospital menus offered this information, we estimated the carbon footprint and SFA content of each dish based on the ingredient amounts reported in similar recipes drawn from a popular UK recipe site.
— In our earlier proof-of-concept intervention, we used a 2-alternative forced-choice (2AFC) task to assess students’ relative preference for every dish across weekly menus. As noted above, for each hospital, we collected preference data from residents in the same region (free-living individuals), rather than from actual hospital inpatients.

## 2. Methods

### (a) Selection and extraction of weekly menus

The UK is commonly split into 12 regions: Wales, Scotland, Northern Ireland and nine regions in England (London, North East, North West, Yorkshire, East Midlands, West Midlands, South East, East of England and South West). Within each region, we convenience sampled a NHS hospital that made a weekly inpatient dining menu publicly available (details of the hospitals can be found in table 1). Aligning with the first aim of the study—to evaluate the suitability of applying our approach to hospital menus (§1)—we carefully reviewed the menu from each site at the outset of the analysis. Of the 12 menus (H01–H12), one (H06) was deemed unsuitable, and we explain our reasoning for this in §3.

**Table 1.** Names and sizes (number of beds) of the 12 hospital sites across the UK.

site number	UK region	site name	number of beds
H01	North East, England	County Durham and Darlington NHS Foundation Trust	1198
H02	North West, England	Wrightington, Wigan and Leigh Teaching Hospitals NHS Foundation Trust	758
H03	Yorkshire and the Humber, England	United Lincolnshire Hospitals NHS Trust	942
H04	East Midlands, England	Nottingham University Hospitals NHS Trust	1700
H05	West Midlands, England	University Hospitals Coventry and Warwickshire	1005
H06	East of England	Norfolk and Norwich University Hospital	1200
H07	London, England	St George’s University Hospitals NHS Foundation Trust	1300
H08	South East, England	Ashford and St Peter’s Hospitals NHS Foundation Trust	575
H09	South West, England	University Hospitals Bristol and Weston NHS Foundation Trust	1363
H010	Wales	Cardiff and Vale University Health Board	1779
H011	Scotland	NHS Great Glasgow and Clyde	6000
H012	Northern Ireland	Royal Victoria Hospital, Belfast	611

Consistent with our previous proof-of-concept study [6], for the remaining 11 menus, we extracted all dishes that were served at a mealtime on Monday through Friday. All hospitals served one large hot meal per day but the time of day differed (some serving a main meal at lunchtime and lighter options in the evening, and others the reverse of this). Thus, depending on when a main meal was served, we selected dishes from either a lunch or a dinner menu. Additionally, the total number of menu options differed across sites. To standardize the application of our modelling across sites, to align with our previous study [6] and ultimately to ensure that results from both studies are mutually comparable, for each site, we extracted the first three dishes available on each day (165 dishes in total; 11 sites × 3 dishes × 5 days) to form a baseline dataset. In two sites (H07 and H08), there was 1 day where only 1 meat option was offered. Here, the corresponding meat dish was selected along with the two vegetarian dishes offered. When more than three dishes were listed in a menu, the first two meat dishes were selected, along with the first vegetarian dish. However, this was only necessary in two hospitals.

### (b) Calculation of saturated fatty acid content and carbon footprint

Unlike the proof-of-concept study that was carried out within our own university, the hospital menu data were not accompanied by recipe information. To obtain an approximate estimation of SFA content and carbon footprint, we needed to estimate ingredient amounts used for each dish. Here, we mirrored an approach commonly employed to screen text when conducting a systematic literature review. Specifically, two independent reviewers accessed the BBCGoodFood recipe website (https://www.bbcgoodfood.com), which provides detailed information about ingredients and quantities needed to produce a wide range of dishes cooked in the UK. Because BBCGoodFood hosts recipes submitted by both professional and amateur chefs, a popular dish will often have several versions of a recipe, sometimes under slightly different names. Therefore, each reviewer was tasked to identify their top three best-fitting recipes for each of the 165 dishes.

After this recipe-matching task, the reviewers conferred to agree on the best-fitting recipes. When both reviewers selected the same first choice, this recipe was automatically selected. In the remaining cases, agreement was met following discussion, and, in a few cases, a third reviewer assisted.

Next, for each dish, we extracted a list of ingredients and amounts from the BBCGoodFood website and uploaded them to Klimato (https://app.klimato.se). Klimato is an online recipe management platform that we have used previously to calculate the carbon footprint and SFA content of dishes and it was selected for the robustness of its background computational processes, which have been externally reviewed [6].

Some recipes included the instruction ‘spice/season to taste’. Because the impact of arbitrary spicing is difficult to quantify (and likely to be trivial), we excluded ‘to taste’ ingredients from our recipe entry. In addition, some recipes incorporated a side dish with a main component (e.g. rice served with curry). Here, to ensure the interchangeability (for swapping) between 15 dishes and also to maintain consistency with our previous work [6], we excluded side components from our calculation.

### (c) Weekly menu optimization

#### (i) Overall strategy

Figure 1 (panel A) shows how our approach works conceptually at the individual consumer level. However, an added complexity in this real-world implementation is that food choices can differ, both across individuals and across regions. To account for regional differences in food preference, for each of the 11 regions, 50 locally residing participants (i.e. potential inpatients) assessed their preference for 15 dishes (3 dishes × 5 days) listed on a menu in their regional hospital. Rather than optimizing at an individual level, our approach generates a single menu that is optimized to deliver maximum benefit across individuals; carbon footprint and SFA intake were minimized across 50 people across 5 days, accounting for heterogeneity in food preference. To achieve this, first, for each hospital, we estimated the number of times each dish would be selected under every possible permutation of a weekly menu formed from the 15 dishes (details below). Then, for each permutation, we calculated the associated weekly carbon footprint and SFA intake, and based on this information, we selected menus that minimized one or both variables.

#### (ii) Meal preference assessment

Following the process adopted in the proof-of-concept study [6], we assessed meal preference using an online 2AFC task. Participants were shown two buttons side-by-side, each labelled with the name of a dish. Each dish (*n* = 15) was paired with every other dish, rendering 105 trials in total. The order of trials was randomized and participants were asked to select which meal they would choose. This type of online food evaluation study is commonly used in psychology; see, for example, Brunstrom & Schatzker [19], Buckley *et al.* [20] and Perszyk *et al.* [21].

Participants (*n* = 550; 11 hospital regions with 50 participants per region) were recruited using Prolific (https://www.prolific.com). To ensure that participants were living in the same region as the hospital, we used Prolific’s demographic pre-screening function. The same function was also used to pre-select participants who consumed meat, and we applied quota sampling to ensure an even split between males and females. For each region separately, demographic details can be found in table 2.

**Table 2.** Demographics of participants (*n* = 548) from the 2AFC data collections across 11 UK regions.^a^

UK region	male	female	age (s.e.)
East Midlands, England	26	24	42.7 (2.1)
London, England	25	24	37.2 (1.5)
North East, England	25	25	37.9 (1.8)
North West, England	24	25	39.6 (2.5)
Northern Ireland	25	25	38.9 (1.9)
Scotland	25	25	40.0 (2.0)
South East, England	26	24	42.6 (2.1)
South West, England	25	25	39.9 (1.9)
Wales	26	24	40.4 (1.9)
West Midlands, England	25	25	39.6 (1.9)
Yorkshire and the Humber, England	25	25	40.2 (2.0)

^a^ Demographic information is missing for two participants.

Participants were informed that the purpose of the study was to evaluate preference for different meals to determine whether strategically swapping dishes can help make menus healthier and more sustainable. After providing consent, participants completed the choice task, were offered debrief information, provided final consent and were remunerated with £1.20 for completing the study (£9 per hour, mean study completion time: 5 min and 32 s). Finally, for each participant separately, we obtained the ranking among the 15 dishes according to the number of times each was selected across the 105 trials. The study was hosted on the online platform Pavlovia (https://pavlovia.org) and ethical approval was granted by the University of Bristol Faculty of Life and Health Sciences Ethics Committee (approval number 19577).

#### (iii) Menu ranking and selection

Our approach to modelling the impact of strategic menu optimization on weekly carbon footprint and SFA intake was based on our previous work [6]. Briefly, there are 1 401 400 unique combinations comprizing five groups of three dishes (i.e. a weekly menu), without considering the order of appearance among the five groups. This was then reduced to 113 400 permutations after a ‘vegetarian constraint’ was applied, meaning that a minimum of one vegetarian dish must be offered each day. For sites H07 and H08, where the original menu included a day on which two vegetarian dishes were offered, the number of permutations satisfying the vegetarian constraint was 340 200.

For each of the 11 sites, we then used the preference ranking data from the online 2AFC task (§2c(ii)) to calculate the weekly carbon footprint (g CO_2_e) and SFA intake (g) associated with each menu combination. For each participant under each of the 113 400 different menu combinations, we used these ranks to identify the five dishes that each participant would most likely choose across the 5 days. Finally, we combined the results across participants to account for heterogeneity and computed the average weekly carbon footprint and SFA intake across the 50 participants. This process was conducted separately for each site.

To quantify the efficacy of strategic menu design, for every potential menu combination, we took the baseline menu (i.e. the starting menu selected by the catering providers) and calculated the differences (%) in predicted weekly carbon footprints and SFAs, both independently for each variable and jointly for both variables under equal weights. A larger (negative) difference indicates a greater reduction relative to the baseline, and from this, we selected the menus that generated the greatest weekly reductions as the optimal strategies.

## 3. Results

As noted in §2.1, and aligning with the study’s first aim, the first step in our analysis was to assess the feasibility of our dish-swapping approach at each site. An examination of the original menus revealed that the method was suitable for 11 out of 12 hospitals. The menu in one hospital (H06) comprised only six unique dishes across the week (the same dishes were repeated either twice or three times), greatly limiting the number of unique menu combinations that could be generated (12 compared with 113 400). In turn, this substantially constrained the scope for reductions, especially when jointly targeting both outcome variables.

**Figure 2.**
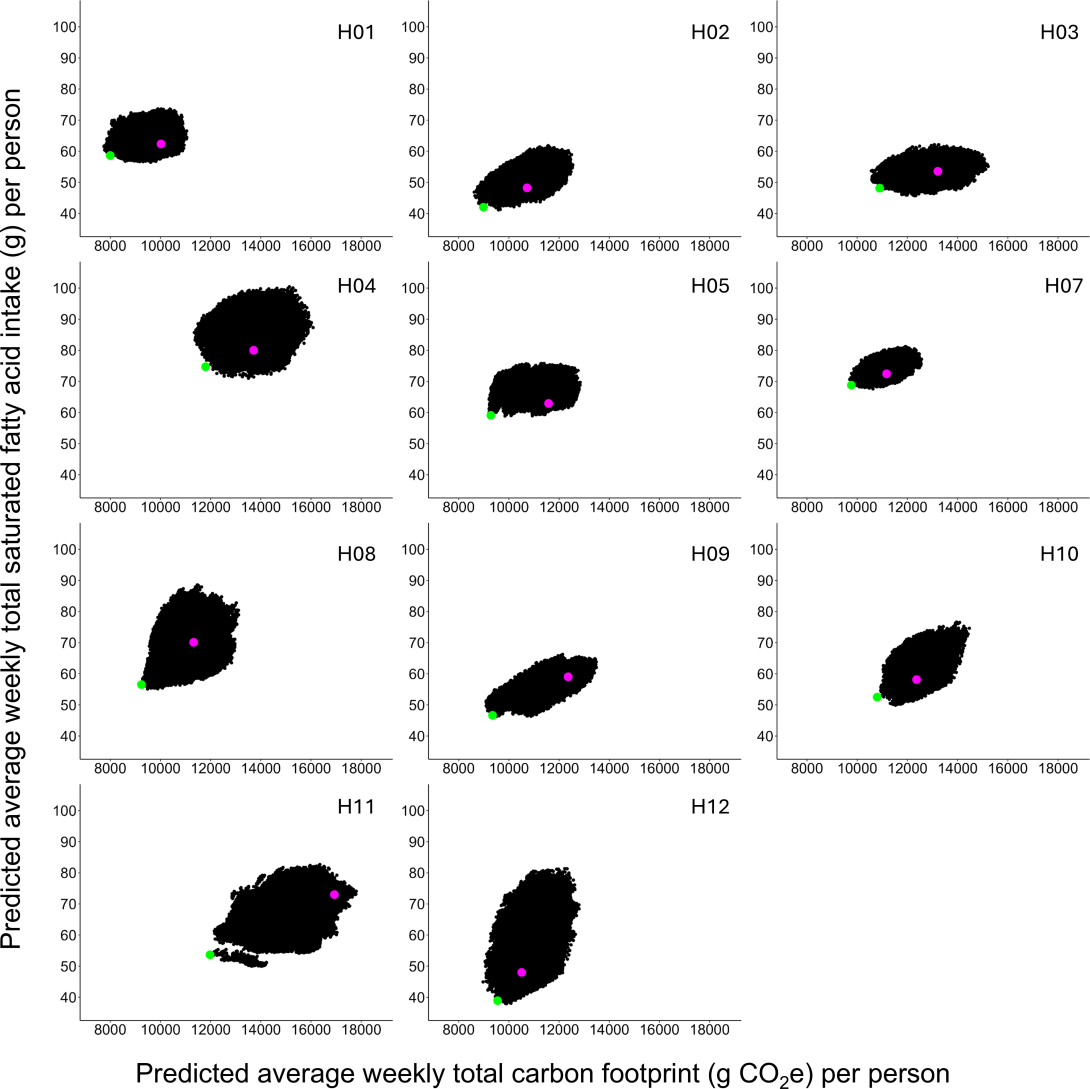
A panel plot showing the visualization of the predicted average weekly total carbon footprint (g CO_2_e) and SFA intake (g) associated with each of the possible menu combinations for one individual for each of the 11 sites where menu swaps were possible. In each panel, the pink circle indicates the predicted values associated with the baseline menu, while the green circle indicates the predicted values for the menu that would minimize both carbon footprint and SFA intake*.*

Across the remaining 11 hospitals, when minimizing carbon footprint as a sole target variable, we achieved a predicted reduction of 12.7–29.3%. When minimizing only SFA intake, the corresponding reductions were between 6.5% and 31.5%. Finally, following joint minimization of carbon footprint and SFA intake, we predicted reductions ranging from 9.1 to 29.3% and 5.0 to 26.5%, respectively. Table 3 reports the predicted reductions at each hospital site. Figure 2 shows the distribution of predicted carbon footprints and SFA intakes for every possible menu combination, and for each of the 11 hospitals, separately.

**Table 3.** Predicted reductions in weekly carbon footprint (CF, g CO_2_e) and intake of SFA (g) achieved by strategic menu-item swapping in the 11 sites where menu swaps were possible.

site	mean weekly CF per person	mean weekly SFA per person	% reductions when targeting a single variable		% reductions when minimizing across two variables	
			CF	SFA	CF	SFA
H01	9477.7	64.7	−22.6	−9.5	−20.2	−6.0
H02	10 723.4	51.3	−19.6	−14.7	−16.2	−12.9
H03	12 951.8	54.3	−20.0	−14.6	−17.5	−10.1
H04	13 789.0	86.1	−17.2	−11.2	−14.0	−6.6
H05	11 077.3	66.4	−20.5	−6.5	−19.9	−6.1
H07	11 048.8	74.2	−13.2	−6.7	−12.6	−5.0
H08	10 995.9	70.0	−18.4	−21.4	−18.4	−19.4
H09	11 475.0	56.4	−26.4	−21.6	−24.4	−20.9
H10	12 526.9	63.0	−12.7	−14.1	−12.7	−9.7
H11	15 264.1	67.5	−29.3	−31.5	−29.3	−26.5
H12	10 809.1	57.4	−14.7	−20.9	−9.1	−18.9

We note that the scope for reduction in our variables of interest differs across sites. With our approach, the magnitude of reduction primarily depends on two factors: (1) the range of variation in targeted variables across every possible menu combination (indicated by the size and shape of each ‘island’ in figure 2); and (2) the location of the baseline menu within each island—both horizontally and vertically (shown as pink circles in figure 2). For example, inspection of figure 2 shows that the distribution of the predicted carbon footprint for H12 is relatively narrow and, within this range, the baseline carbon footprint is already low. In combination, these two factors limit the potential to generate an improvement for this particular target variable. In contrast, for H03, the distribution of carbon footprint is relatively wide, and the distance between the baseline carbon footprint and the predicted under the optimum menu is relatively greater. Thus, when optimizing alongside SFA intake, in H03, our approach predicted a 17.5% reduction in carbon footprint, whereas in H12, we predicted only a 9.1% reduction.

## 4. Discussion

### (a) Strengths of strategic menu swapping

Using the strategic menu optimization approach, our proof-of-concept study in a university canteen demonstrated substantial reductions in carbon footprint and SFA intake. To assess the approach’s efficacy in another public procurement setting, here, we applied it to inpatient menus at hospitals across the UK. The results suggested that, with standardized weekly menus (three main meal options per day, served either at lunch or dinner for 5 consecutive days), our approach has a similar level of potential to deliver meaningful benefits, without the need to change recipes or include new dishes. In so doing, this work has provided further early-stage evidence supporting the application of our approach.

A strength of our approach is that it can be applied alongside strategies based on reformulation. Some of these are captured in the work of other projects that form the TUKFS Network. For example, to reduce climate impact, FixOurFood is exploring ways to reformulate individual dishes in school canteens [22], while Mandala is doing the same for inpatient hospital meals [23]. After reformulation, our approach can be applied to meet additional environmental and health targets. At the ingredient level, the Hi-Fi Bread project is developing a high-fibre white loaf bread [24], and the Pasture to Plate Team are developing a novel grass-derived food product [25]. In both cases, these novel foods might be combined with our approach to effect a dual positive outcome.

Furthermore, although we minimized across carbon footprint and SFA, hospitals could choose other variables of interest, such as salt or sugar intake, and they could also consider maximizing variables such as fibre intake or operational profit. In this regard, we note that any attempt to optimize two or more variables will depend on their correlation structure, and in cases where it is negative and strong, joint optimization will be problematic because a reduction in one will necessarily require an increase in the other.

Finally, with our approach, catering providers are not required to change their recipes or procurement practices, and because no meal is removed from the weekly menu, consumers can continue to select and enjoy their most preferred dish. Nonetheless, to realize these benefits, practical challenges would need to be met, and these are considered in the sections that follow.

### (b) Limitations of the present study

Although hospitals were sampled from different geographic regions, we convenience-sampled hospitals for which menus were publicly available, meaning that they are not necessarily representative. Nevertheless, all sites are NHS hospitals, which subscribe to a common set of standards for inpatient meal provision [26]. A similar limitation on representativeness also relates to our selection of participants who completed the online choice task. We ensured an equal balance of males and females, and that for each hospital, respondents were living in the same region. However, our participants were likely younger than individuals typically admitted for adult inpatient care [27], and future work should investigate whether the accuracy of predicted outcomes is impacted by a mismatch between the age of participants providing preference data and that of hospital inpatients.

As noted in §1, to validate and directly compare these outcomes with our previous work [6] and to achieve an early estimate of the potential benefits of our approach in a hospital setting, we (1) limited our modelling to dishes offered on weekdays and to three main meals per day, and (2) estimated recipe information from a popular online resource. With regard to the former (point 1), this decision was made to align the menu structure of the NHS hospitals with our university canteen menus (three evening meal options per day). This was only necessary in two cases, and in the remaining 9 of 11 hospitals, three main dishes were already offered to patients. Further work will be needed to quantify the effects of menu-item swapping when four or more dishes are offered and, more generally, to determine the minimum requirements for the initial menu structure to make it suitable for our approach. With the latter (point 2), our decision to use an online recipe site may have introduced errors to our calculation of carbon footprint and SFA content. However, if they take the form of a systematic overestimation of the carbon footprint or SFA content of hospital meals, then we note that our findings would remain unchanged. This is because we have reported % reductions (i.e. relative reductions), which are unaffected by systematic under- or over-estimation of these target variables under both baseline and optimized menus.

Finally, our modelling did not account for special dishes (e.g. easy eating, gluten-free or low sodium) and side dishes (e.g. mashed potatoes and rice). Although the ordering frequency of special dishes and the contributions of side dishes to carbon footprint and SFA intake are both likely to be smaller than the main dishes studied herein, we acknowledge that, to obtain estimates of per cent reductions in all cases and for entire meals, more extensive modelling would be required.

### (c) Practical challenges of real-world implementation

To realize the real-world application of our approach in hospitals or another public procurement setting, further investment and research are needed. First, we would recommend developing a user-friendly interface that would enable catering providers to use our modelling algorithm without coding capability. It might also be helpful to consider whether other forms of preference information might be sourced that obviate the need to collect preference data directly from patients and other diners, for example, via sales data or related indices of popularity. More generally, to promote real-world application, we see a need to engage with key stakeholders (e.g. nutritionists, catering providers and hospital administrators) to understand their perceptions of feasibility, acceptability and costs, and to conduct a quantitative cost–benefit analysis. In this regard, and as noted above, we see the potential to mitigate any impact on operating costs by implementing menu swaps with operational profit as one of the target variables.

While we observed reductions in our outcomes of interest across 11 out of 12 hospitals, it should be noted that our approach might not be well-suited to every menu, especially when too few or too many daily menu options are offered. Our approach is unlikely to lend itself to menus where dishes frequently repeat across a week (i.e. only six unique dishes are offered across 5 days) as this significantly limits the number of unique menu combinations that can be generated. Conversely, it may be possible to apply our approach when four or more dishes are offered on a daily menu. However, this could increase the computational time substantially, and it remains unclear how this might affect the potential for reductions in target variables. Accordingly, future research is needed to quantify the effects of strategic menu-item swapping on a range of non-standard menu types.

### (d) Summary and next steps

Strategically swapping menu items across a weekly hospital menu has the potential to reduce its food carbon footprint and intake of SFA by an estimated 9.1–29.3% and 5.0–26.5%, respectively. To validate these predictions, we plan to trial our approach in a small number of NHS hospitals. More generally, this will provide a useful opportunity to characterize barriers to, and enablers of, practical implementation, including those related to logistical, financial and labour constraints, as well as the level of uncertainty associated with the model. If this process garners evidence supporting the application of our methods, then our longer-term plan includes its broader implementation in other catered environments, including schools, care homes, workplace canteens and commercial food outlets, accompanied by estimates of population-level impacts when fully upscaled.

## Data Availability

The data and code can be found in electronic supplementary materials [28].
